# Spinal fracture reveals an accident episode in *Eremotherium laurillardi* shedding light on the formation of a fossil assemblage

**DOI:** 10.1038/s41598-022-08107-1

**Published:** 2022-03-08

**Authors:** Fernando H. de S. Barbosa, Hermínio I. de Araújo-Júnior, Isadora da Costa, André Vieira de Araújo, Edison Vicente Oliveira

**Affiliations:** 1 Departamento de Estratigrafia e Paleontologia, Faculdade de Geologia, Universidade do Estado do Rio de Janeiro (UERJ), Rua São Francisco Xavier, 524, Maracanã, Rio de Janeiro, RJ 20550‑013 Brazil; 2 Programa de Pós-Graduação em Geologia, Universidade Federal do Rio de Janeiro (UFRJ), Avenida Athos da Silveira Ramos, 274, Ilha do Fundão, Rio de Janeiro, RJ 21941-916 Brazil; 3 Programa de Pós-Graduação em Geologia, Faculdade de Geologia, Universidade do Estado do Rio de Janeiro (UERJ), Rua São Francisco Xavier, 524, Maracanã, Rio de Janeiro, RJ 20550‑013 Brazil; 4 Laboratório de Paleontologia (PALEOLAB), Departamento de Geologia, Centro de Tecnologia e Geociências, Universidade Federal de Pernambuco (UFPE), Av. Acadêmico Hélio Ramos, s/n, Cidade Universitária, Recife, PE 50740-530 Brazil

**Keywords:** Palaeontology, Palaeoecology

## Abstract

The Toca das Onças cave is one of the most important Quaternary mammal deposits of Brazil. Two different hypotheses have been proposed to explain the preservation mode of its skeletal remains: either the animals climbed down into the cave, or it could have functioned as a natural trap. Evaluation of pathological modifications on three articulated vertebrae of a single adult giant ground sloth *Eremotherium laurillardi* reveals a particular type of bone fracture caused by compressive force on the vertebral column, which split the vertebral bodies in the sagittal plane. This diagnosis suggests that the animal accidentally fell into the cave, in accordance with the second hypothesis proposed to the incorporation mode of skeletal remains into the cave.

## Introduction

The natural world is a tough place, where wild animals need to constantly adapt to different biotic and abiotic conditions to survive. In past animals, evidences of this “fight for survival” can be revealed through different types of analyses of bones preserved in the fossil record (e.g., ichnologic^1^; and isotopic^2^). An interesting category of evidence about the daily life of ancient animals are bone trauma – any break in the bone continuity caused by a force or mechanism extrinsic to the body^3^. This type of bone injury is able to reveal several paleoecological information such as intra and interspecific conflicts (e.g.,^4,5^), occupational activities (e.g.,^6,7^) and accidents^8^.

Despite its paleobiological significance, bone fractures in past animals remain underexplored when compared with other types of paleopathological conditions (e.g., arthritis^9^, and infections^10^), especially concerning the Pleistocene megafauna from South America. Recently, few cases of fractures have been described to this fauna, including a case in the ground sloth *Nothrotherium maquinense*^11^ and two independent cases in the giant ground sloth *Eremotherium laurillardi*^12,13^. Regarding the giant ground sloths, the record of complete and articulated *Eremotherium* skeletons in the Toca das Onças site has enabled inferences about the death and mode of incorporation of these remains into this cave deposit^14,15^.

This paper provides a new case of bone trauma in *Eremotherium laurillardi*, the only Pan-American megatheriine giant ground sloth and one of the taxa most often found in the Brazilian Quaternary fossil deposits^16,17^. In addition to detailing the fractures’ origin mechanism, we discuss how this particular case of bone injury can be used to shed light on the possible cause of death of the individual studied, on the incorporation of its bone remains into the taphocoenosis of the cave, and on the depositional aspects of caves.

## Results

**Figure 1:**
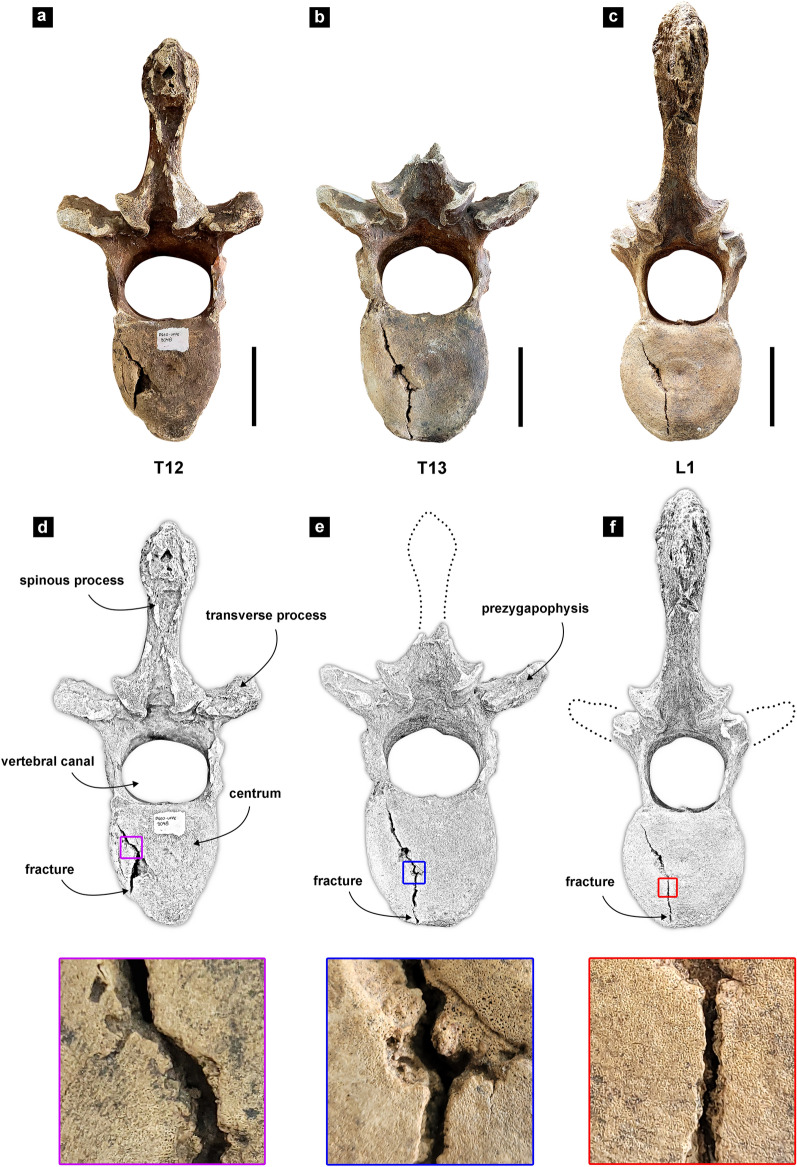
Thoracic T12 and T13, and lumbar L1 vertebrae of *Eremotherium laurillardi*. (**a–c**) Photography of T12, T13 and L1 in caudal view, (**d**–**f**) schematic drawing of vertebrae in caudal view and close-up view of the traumatic lesions. Scale bars: 10 cm.

The three vertebrae investigated (DGEO UFPE 5769, DGEO UFPE 7167 and DGEO UFPE 9048) belong to the same individual and show lesions with similar appearance, location, and size. All lesions are in the caudal endplate of each vertebra near the left edges of the vertebral bodies running in the dorsoventral direction with a gently oblique position (Fig. 1). Each lesion appears as a deep and narrow bone discontinuity which has its gap widening at the midpoint before narrowing again, something that is most noticeable in the vertebra DGEO UFPE 9048 (Fig. 1a). Each lesion has approximately the following measurements of length: (1) 13.3 cm (12th thoracic vertebra, DGEO UFPE 9048); (2) 13.8 cm (13th thoracic vertebra, DGEO UFPE 7167); and (3) 8.4 cm (1st lumbar vertebra, DGEO UFPE 5769). In addition, it is possible to observe that all lesions have smooth and rounded edges (see images in detail in Fig. 1). There is no macroscopic evidence of new bone formation on the surface of the vertebrae.

## Discussion

Since the bone discontinuities noted in the three vertebrae analyzed show no clear sign of bone overgrowth, it is pivotal to rule out the possibility that we are dealing with preservation damages before proposing an accurate diagnosis for the lesions. The close-up view examination of the abnormalities shows that their edges have clear signs of smoothing and rounding (Fig. 1), which represent important evidence of osteoblastic activity^18,19^. Additionally, the similar color of the cortical damage and normal bone can be used as secondary evidence to rule out post-mortem processes as a possible origin of the alterations, since recent destructive processes are lighter than the rest of the bone^19^. Therefore, as taphonomic processes can be ruled out, the pointed evidence strongly suggests that the discontinuities observed are of pathological origin. More specifically, these breaks found in all three vertebrae are indicative of bone fracture.

Based on fracture analysis criteria applied here^20^, which consider the location and morphological pattern of the fractures, we classified the fractures noted in all vertebrae as traumas belonging to Type A (vertebral body compression), Group A2 (split fractures), and subgroup A2.1 (sagittal split fracture). This diagnosis implies that the traumatic episode was likely caused by a compressive force on the vertebral column, which split the vertebral bodies in the sagittal plane. This type of injury is considered stable—i.e., the fracture does not have a tendency to displace after reduction—and neurological deficit is uncommon^20,22,23^. Although stable traumas cause only moderate pain, without generating significant movement limitations^20^, the *Eremotherium* individual here analyzed died with unhealed bones, as there is no evidence of callus formation.

The absence of other skeletal signs that point to the presence of another type of disease concomitantly to the fractures allows us to reject the possibility that they have been generated as a result of a pre-existing disease (e.g., infection, neoplasm). We also consider that the vertebral injuries were not caused by repetitive force (stress fractures) because this type of injury is commonly characterized as a nondisplaced line or crack in the bone, called hairline fracture^3^. Those refer to situations where the broken bone fragments are not visibly out of alignment and exhibit very little relative displacement^21^. Although the *Eremotherium* vertebrae fractures’ can be described as nondisplaced, they also have a noticeable gap between their edges that is mostly narrow with wider parts in the middle, something found in split fractures^20^ but that is not characteristic of hairline fractures. Lastly, the subgroup C1.2.1 (rotational sagittal split fracture) might be a source of confusion due to similar morphological pattern with subgroup A2.1 (sagittal split fracture). However, in subgroup C1.2.1 there are compressive and rotational forces acting simultaneously, producing total separation into two parts^20^, which clearly did not occur in the vertebrae analyzed here.

In humans, compression fractures are most commonly caused by osteoporosis, although infection, neoplasm and trauma can also be etiological factors^23–25^. However, as aforementioned, the absence of other pathological skeletal marks is an important characteristic to take note as it serves to disregard the possibility of the fractures’ genesis to be secondary to another pathology. As such, in this case, osteoporosis, infection and neoplasm are unlikely etiologies. On the other hand, a compression fracture in a healthy individual is commonly generated after a severe traumatic event such as a fall from great height^23,26^. This scenario seems to better explain the origin of the vertebral fractures in the case of the *Eremotherium* ground sloth herein studied.

**Figure 2:**
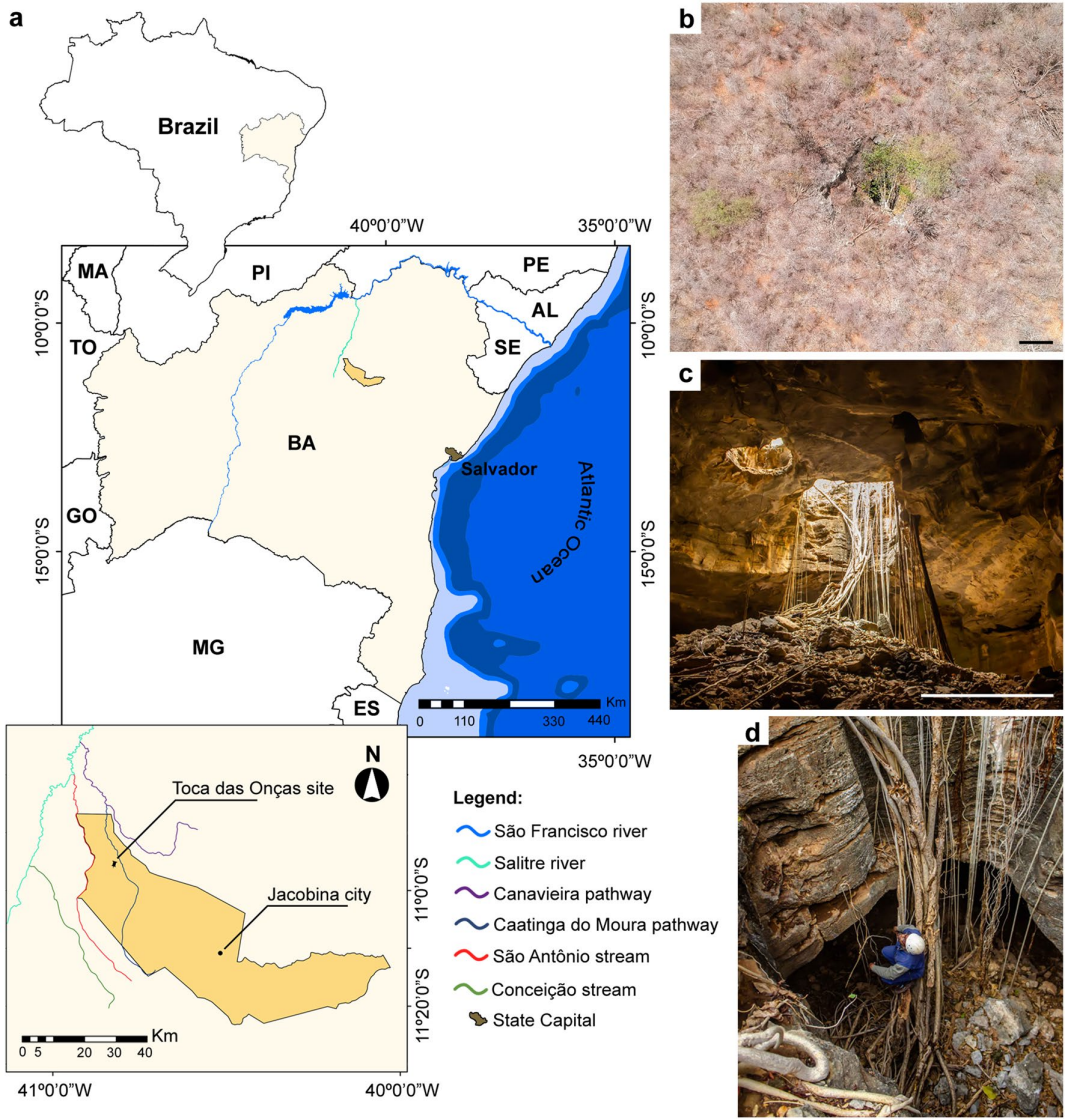
Location map of the Toca das Onças site and images of the cave. (**a**) Detail of the location, (**b**) cave entrance area view, (**c**) view from inside the cave, (**d**) Cave entrance detail. Scale bars 10 m in (**b**) and 5 m in (**c**). This figure was generated by Adobe Photoshop CS6 software (https://www.adobe.com/br/products/photoshop.html).

**Figure 3:**
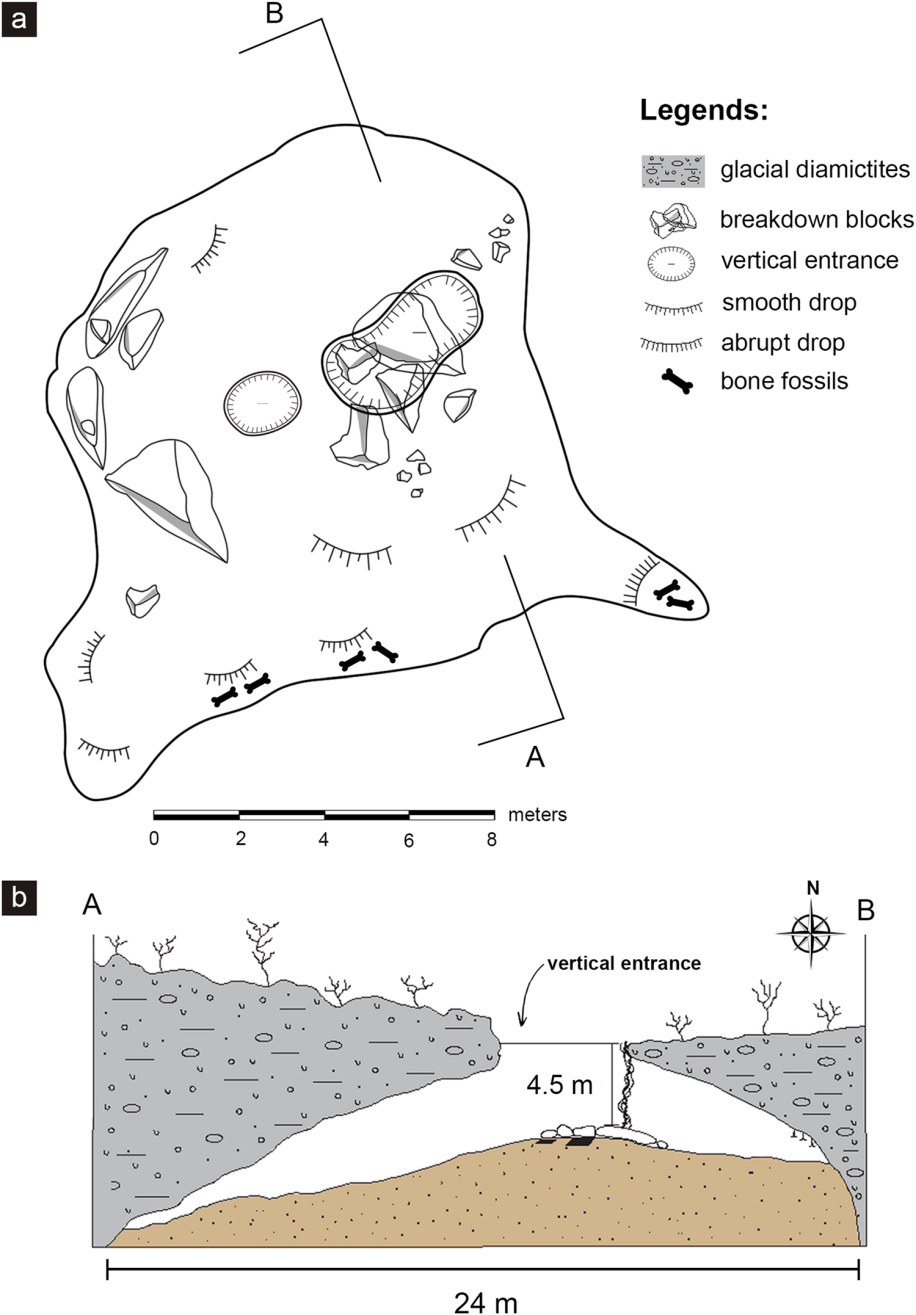
Schematic representation of the Toca das Onças site. (**a**) Ground plan of the cave illustrating its morphology and dimension, (**b**) Cross-section illustrating the abyss-shaped entrance.

The three fractured vertebrae were recovered in the Toca das Onças site (Fig. 2), a small cave considered as one of the richest paleontological sites of the Brazilian Quaternary^15^. Two complete skeletons of *Eremotherium laurillardi* and fragments belonging to at least thirteen other individuals, together with several other bones assigned to different smaller species are known to this cave^14^. It comprises of a single dry chamber that can only be entered through vertical entrances approximately 4.5 m high (Figs. 2b–d and 3). Two different hypotheses concerning the depositional process of Toca da Onças were previously proposed: (1) the animals climbed down into the cave in search of water^14^; or (2) due to the vertical character of the cave entrance, it could have functioned as a natural trap where animals accidentally fell into the cave^15^.

The first hypothesis would indicate that the animal fell into the cave during an attempt to climb down. However, there is no report in the literature indicating that *Eremotherium laurillardi* could have been a climbing animal. In addition, the vertical morphology of the cave entrance would be a limiting factor for climbing behavior (see Fig. 3).

Therefore, based on the type of fracture (compression sagittal split fracture) observed in the three vertebrae of *Eremotherium* as well as the inferred origin mechanism (fall from a great height), the presence of the individual here analyzed in the fossil accumulation of Toca das Onças is more likely explained by the second hypothesis. This idea is not particularly new as ‘entrapment due to fall’ has been described as a fossil accumulation mode to several other caves worldwide (e.g.,^27,28^). However, the use of bones fractures as an indicator of fossil accumulation mode is an interesting novelty. Of course, a detailed taphonomic investigation in the Toca das Onças still needs to be conducted in order to accurately interpret the formation of this important Quaternary fossil accumulation from Brazil.

**Figure 4:**
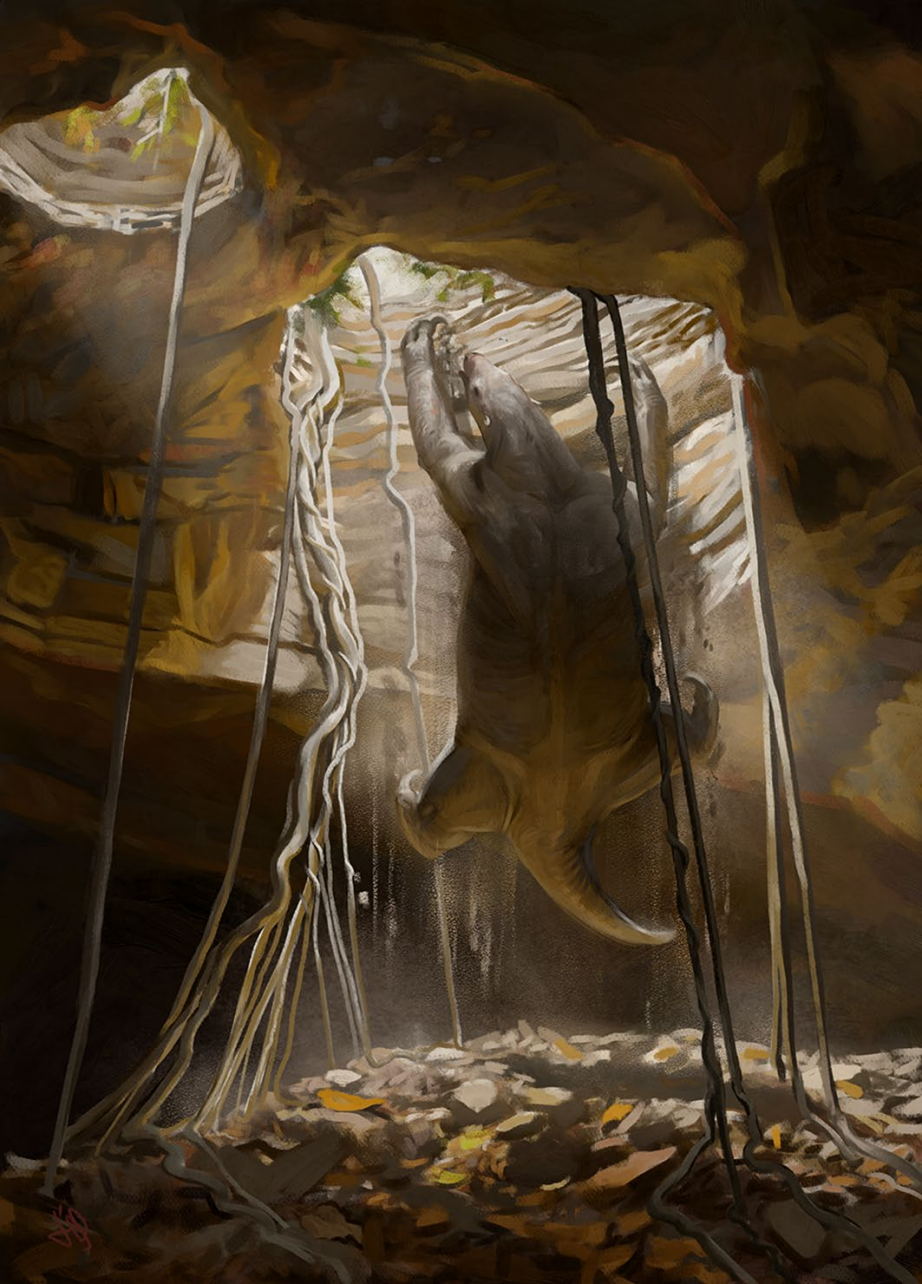
Artistic reconstruction of the suggested fall of the individual *Eremotherium laurillardi* into the cave. Artwork by Júlia d’Oliveira.

In sum, we suggest that the animal accidentally fell into the cave, fractured at least three sequential vertebrae (12th, 13th thoracic vertebrae and 1st lumbar vertebra) after the impact on the ground, survived for a while, but succumbed trapped inside the cave without food and water (Fig. 4). Other animals found in the cave, but without signs of bone fracture, may have fallen and not fractured their bones or not survived after the fall, especially the smaller ones. Finally, the proposal of falls to explain the unusual record of giant ground sloth fossils preserving much of its skeleton in caves, as reported for Toca das Onças site, contrasts with the better-documented pattern of skeletal accumulation via hydraulic action.

## Materials and methods

The material herein analyzed consists of three articulated vertebrae of an adult—indicated by the presence of vertebral plates completely fused—individual of *Eremotherium laurillardi*, as follows: (1) the twelfth thoracic vertebra (DGEO UFPE 9048); (2) the thirteenth thoracic vertebra (DGEO UFPE 7167); and (3) the first lumbar vertebra (DGEO UFPE 5769). The specimens were recovered from the Toca das Onças site (WGS84-UTM 300,604 m E; 8,791,416 m N), a small cave deposit located at the village of Caatinga do Moura, municipality of Jacobina, State of Bahia, Brazil (Fig. 2) and are housed in the Paleontological Collection of Geology Department of the Universidade Federal de Pernambuco (DGEO UFPE), Recife city, State of Pernambuco, Brazil.

The Toca das Onças site was developed in the Neoproterozoic dolomitic rocks of the Salitre Formation, Una Group. The cave has a single vertical entrance with a drop of approximately 4.5 m and a total area of 16 m^2^^29^. It consists of a single chamber with a linear development of 23 m and a volume of 63 m^3^^29^.

Each vertebra was macroscopically examined to characterize the pathological changes. We adopt here the fracture classification system proposed by Magerl et al.^20^ to describe the lesions studied. This classification system was created specifically for thoracic and lumbar vertebral fractures and is based on morphological patterns that can be divided in a set of three main categories of lesions (Type A, Type B and Type C). Each category reflects the effect of a force and the mechanism that was involved in the origin of the type of lesion and its subtypes. The system presents a hierarchy of types, groups and subgroups that are ranked according to its progressive severity and are accompanied by detailed morphological features (see^20^ for more details). The length of each fracture was measured using the open-source platform Fiji^30^.

## Data Availability

All data analyzed during this study are included in this work.

## References

[1] Araújo-Júnior HI, Barbosa FHS, Silva LHM (2017). Overlapping paleoichnology, paleoecology and taphonomy: Analysis of tooth traces in a Late Pleistocene-early Holocene megafaunal assemblage of Brazil and description of a new ichnotaxon in hard substrate. Palaeogeogr. Palaeoclimatol. Palaeoecol..

[2] Tejada JV (2021). Isotope data from amino acids indicate Darwin’s ground sloth was not an herbivore. Sci. Rep..

[3] Lovell NC (1997). Trauma analysis in paleopathology. Yearb. Phys. Anthropol..

[4] Farke, A. A., Wolff, E. D. S. & Tanke, D. H. Evidence of Combat in *Triceratops*. *PLoS ONE* **4**, e4252. 10.1371/journal.pone.0004252 (2009). 10.1371/journal.pone.0004252 PMC2617760 19172995

[5] Mackness BS, Cooper JE, Wilkinson CEC, Wilkinson D (2010). Palaeopathology of a crocodile femur from the Pliocene of eastern Australia. Alcheringa.

[6] Rothschild BM, Laub R (2008). Pedal stress fractures in mastodons. J. Paleopathol..

[7] Brown, C., Balisi, M., Shaw, C. A. & Van Valkenburgh, B. Skeletal trauma reflects hunting behaviour in extinct sabre-tooth cats and dire wolves. *Nat. Ecol. Evol.* **1**, 0131. 10.1038/s41559-017-0131 (2017). 10.1038/s41559-017-0131 28812696

[8] Rothschild, B. & Lambert, H. W. First documentation of a greenstick fracture in the fossil record. Possible gout also noted in *Arkansaurus fridayii*. *Hist. Biol.* **33**, 1349–1351 (2019).

[9] Tomassini RL (2020). Gregariousness in the giant sloth Lestodon (Xenarthra): multi-proxy approach of a bonebed from the Last Maximum Glacial of Argentine Pampas. Sci. Rep..

[10] Barbosa FHS, Porpino KO, Fragoso ABL, Santos MFCF (2013). Osteomyelitis in Quaternary mammal from the Rio Grande do Norte State Brazil. Quat Int..

[11] Barbosa FHS, Porpino KO, Bergqvist LP, Rothschild BM (2017). Elucidating bone diseases in Brazilian Pleistocene sloths (Xenarthra, Pilosa, Folivora): First cases reported for the Nothrotheriidae and Megalonychidae families. Ameghiniana.

[12] Dias EMD, Dantas MAT, Barbosa FHS (2020). Diagnosis of bone diseases in two representatives of the Pleistocene megafauna of Bahia Brazil. Hist. Biol..

[13] Andrade LC (2021). Revealing bone diseases in the Quaternary ground sloth *Eremotherium laurillardi* (Mammalia, Xenarthra). Hist Biol..

[14] Cartelle C, Bohorquez GA (1982). *Eremotherium laurillardi* Lund, 1842: Parte I. Determinação específica e dimorfismo sexual. Iheringia..

[15] Auler AS (2006). U-series dating and taphonomy of Quaternary vertebrates from Brazilian caves. Palaeogeogr. Palaeoclimatol. Palaeoecol..

[16] Cartelle C, De Iuliis G (1995). *Eremotherium laurillardi*: the Panamerican Late Pleistocene Megatheriid Sloth. J. Vert. Paleont..

[17] Cartelle, C., De Iuliis, G. & Pujos, F. *Eremotherium laurillardi* (Lund, 1842) (Xenarthra, Megatheriinae) is the only valid megatheriine sloth species in the Pleistocene of intertropical Brazil: A response to Faure et al., 2014*. C. R. Palevol.* **14**, 15–23 (2015).

[18] Ortner, D. J. *Identification of pathological conditions in human skeletal remains* (Academic Press, 2003).

[19] Waldron, T. *Palaeopathology* (Cambridge University Press, 2009).

[20] Magerl F, Aebi M, Gertzbein SD, Harms J, Nazarian S (1994). A comprehensive classification of thoracic and lumbar injuries. Eur. Spine J..

[21] Chowdhury, A. S., Bhandarkar, S. M., Robinson, R. W., Jack, C. Y. & Liu, T. Detection of hairline mandibular fracture using max-flow min-cut and Kolmogorov-Smirnov distance. In *2011 IEEE International Symposium on Biomedical Imaging: From Nano to Macro* (eds IEEE) 1962–1965 (2011).

[22] Simon, R. R. & Sherman, S. C. *Emergency Orthopedics*. (McGraw Hill, 2010).

[23] Alexandru D, So W (2012). Evaluation and management of vertebral compression fractures. Perm J..

[24] Nevitt MC (1998). The association of radiographically detected vertebral fractures with back pain and function: a prospective study. Ann. Intern. Med..

[25] Gertzbein SD (2012). Thoracic and lumbar fractures associated with skiing and snowboarding injuries according to the AO comprehensive classification. Am. J. Sports Med..

[26] Campagne, D. Fraturas de compressão da coluna. *Manual MSD* https://www.msdmanuals.com/pt-br/casa/lesões-e-envenenamentos/fraturas/fraturas-de-compressão-da-coluna (2021).

[27] Andrews P (1990). Owls, caves and fossils: predation, preservation and accumulation of small mammal bones in caves, with an analysis of the Pleistocene cave faunas from Westbury-sub-Mendip.

[28] Simms MJ (1994). Emplacement and preservation of vertebrates in caves and fissures. Zool. J. Linnean Soc..

[29] Araújo AV, Nogueira EE, Cajado EM (1994). Caracterização do Geossítio Toca das Onças no município de Jacobina, Bahia, Brasil. Scientia Plena..

[30] Schindelin J (2012). Fiji: A. open-source platform for biological-image analysis. Nat Methods..

